# Supplementary material for: Applying Proteomics and Computational Approaches to Identify Novel Targets in Blast-Associated Post-Traumatic Epilepsy
Source: Int J Mol Sci. 2024 Mar 1;25(5):2880. doi: 10.3390/ijms25052880 (PMC10932294; doi:10.3390/ijms25052880)
Supplement: Supplementary file 1 [file ijms-25-02880-s001.zip › ijms-2855581-supplementary.pdf]

Supplementary Materials

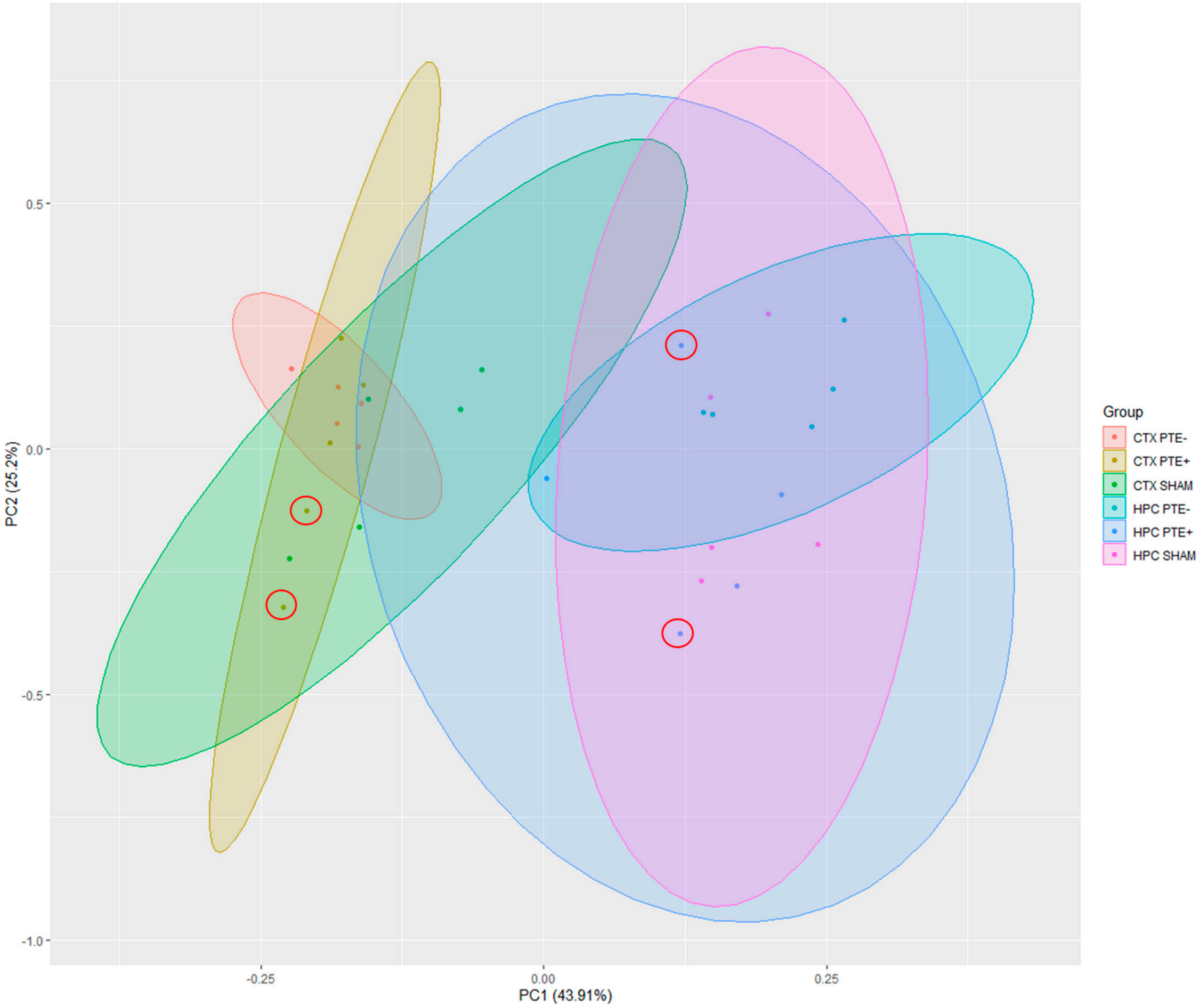

Supplemental Figure S1: PCA analysis of cortical and hippocampal sham, PTE^{+}, and PTE^{-} proteomics. PCA plot shows PTE^{+}, PTE^{-}, and sham injured groups from cortical and hippocampal protein derived from whole proteome. Red circles denote female samples.

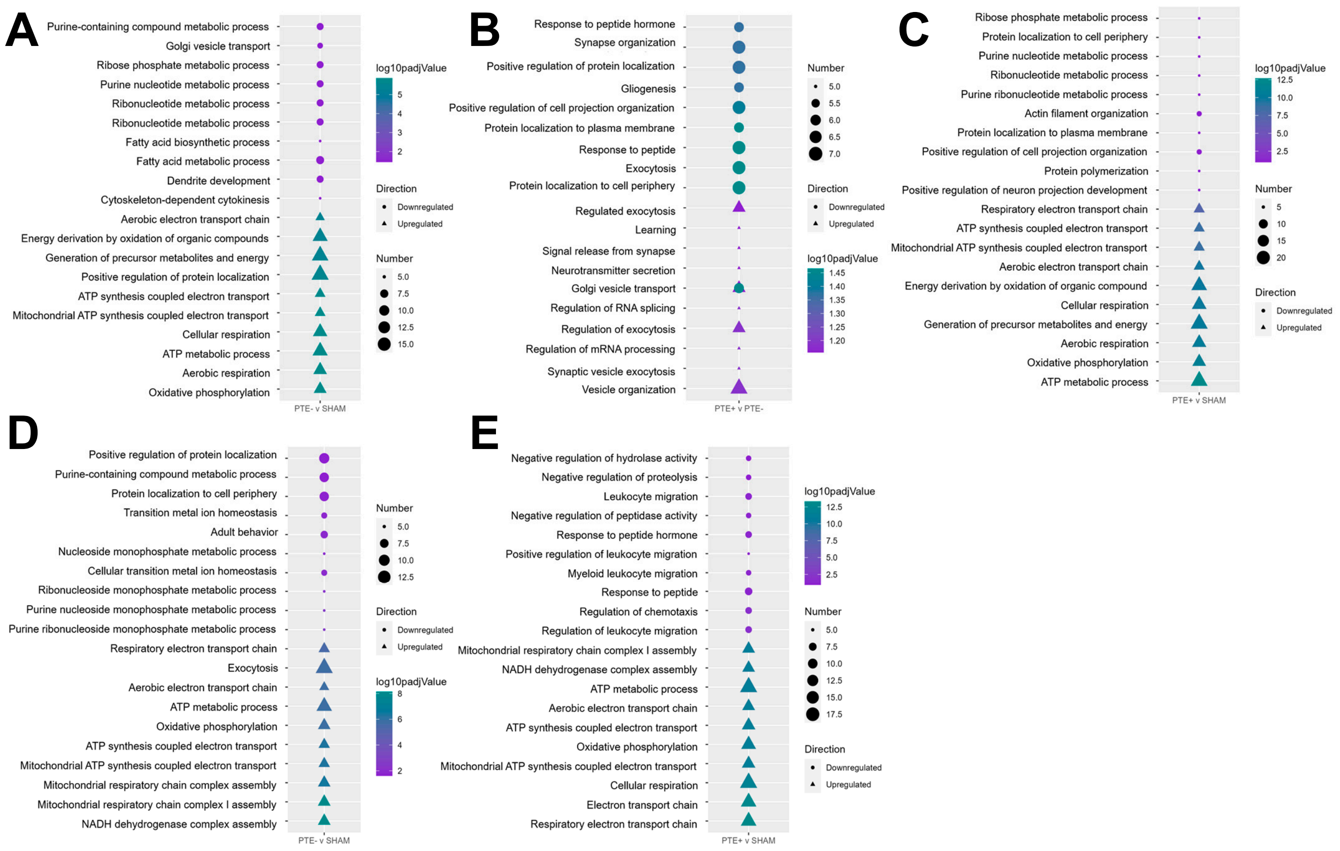

Supplemental Figure S2: Pathways analysis reveals biological process dysregulation among all comparisons of PTE^{+}, PTE^{-}, and sham cortices and hippocampi. Top 10 downregulated (circles) and top 10 upregulated (triangles) GO biological process pathways identified in the whole cortex of (**A**) PTE^{-} vs. sham, (**B**) PTE^{+} vs. PTE^{-}, and (**C**) PTE^{+} vs. sham, or the whole hippocampus of (**D**) PTE^{-} vs. sham and (**E**) PTE^{+} vs. sham comparisons from GO enrichment analysis.
